# Prediction of Apoptosis Protein Subcellular Localization with Multilayer Sparse Coding and Oversampling Approach

**DOI:** 10.1155/2019/2436924

**Published:** 2019-01-30

**Authors:** Xingjian Chen, Xuejiao Hu, Wenxin Yi, Xiang Zou, Wei Xue

**Affiliations:** College of Information Science and Technology, Nanjing Agricultural University, Nanjing, 210095, China

## Abstract

The prediction of apoptosis protein subcellular localization plays an important role in understanding the progress in cell proliferation and death. Recently computational approaches to this issue have become very popular, since the traditional biological experiments are so costly and time-consuming that they cannot catch up with the growth rate of sequence data anymore. In order to improve the prediction accuracy of apoptosis protein subcellular localization, we proposed a sparse coding method combined with traditional feature extraction algorithm to complete the sparse representation of apoptosis protein sequences, using multilayer pooling based on different sizes of dictionaries to integrate the processed features, as well as oversampling approach to decrease the influences caused by unbalanced data sets. Then the extracted features were input to a support vector machine to predict the subcellular localization of the apoptosis protein. The experiment results obtained by Jackknife test on two benchmark data sets indicate that our method can significantly improve the accuracy of the apoptosis protein subcellular localization prediction.

## 1. Introduction

As a basic constituent of organisms, proteins play a critical role in life activities such as metabolism, breeding, growth, and development, especially for the apoptosis protein, which are crucial in the proteomics. Since the functions of an apoptosis protein are closely related to its subcellular location and different kinds of apoptosis proteins can only function in specific subcellular location, it is important to predict the subcellular location of certain apoptosis protein by existing methods, which could not only help us to understand the interactions and properties of apoptosis proteins but also realize the biological pathway involved [1–3]. With the application of high-throughput sequencing techniques and the explosion of sequence data volumes, developing an accurate and reliable computational method to predict apoptosis protein subcellular location has been a great challenge for bioinformaticians, accordingly promoting the development of machine learning in this field [4–8].

By the analysis of research status, the improved directions of using machine learning to predict apoptosis protein subcellular location in the past decade can be roughly categorized into two classes: sequence feature extraction and prediction model [5–10]. Currently the widely used methods for feature extraction are amino acid composition (AAC) [11, 12], pseudo amino acid composition (PseAAC) [13, 14], gene ontology (GO)[15, 16], position specific scoring matrix (PSSM)[17, 18], feature fusion [19, 20], and so on. For example, Zhou et al. used the covariant discriminant function based on Mahalanobis distance and Chou's invariance theorem; combining with traditional AAC feature to predict apoptosis protein subcellular location, the prediction result by Jackknife test on data set ZD98 achieved about 72.5% [21]; Wan et al. proposed GOASVM algorithm based on the information of GO term frequencies and distant homologs to represent a protein in general form of PseAAC and got a high accuracy [22]; Chen et al. used the increment of diversity to fuse N-terminal, C-terminal, and hydrophobic features of apoptosis protein sequences, and the accuracies on ZD98 and CH317 were 90.8% and 82.7%, respectively [23]; Zhao et al. combined the bag of words model with PseAAC method, using K-Means to construct the dictionary of sequence features, and obtained a great predictive effect [24]. At the same time, there are also many efforts for the development of prediction model. For example, Wan et al. proposed an adaptive-decision support vector machine classifier through the annotation information of GO database and realized the prediction of membrane proteins as well as their multifunctional types [25]; Ali et al. extracted the PseAAC features of protein sequences, combining with location voting, k-nearest neighbor and probabilistic neural network to predict the subcellular localization of membrane proteins [26]. Besides, there are also some other prediction models used in this filed such as logistic regression, bayesian classifiers, and long short-term memory [27–29].

In the last decade or so, a recent review [30] pointed that a number of web-servers were developed for predicting the subcellular localization of proteins with both single site and multiple sites [31–36]. In general, proteins can simultaneously exist in multiple sites. In this study, given that the number of multilabel proteins in the existing apoptosis protein database is not large enough to construct a benchmark data set meaningfully in statistics and for the case of multiple locations, the sequence information is more complex and various than single locations, using oversampling approach to copy sequence feature may generate the inaccurate results, so here we did not consider the situation of multilabel proteins.

To summarize the previous research results, it is not difficult to find that the prediction accuracy is relatively low if merely using simple method such as AAC or PseAAC to extract sequence features for classification; as for the other feature extraction methods, like PSSM or feature fusion, though the prediction effect is better, the extraction process is too complicated and time-consuming for practical application. Given that many former researches have proved that support vector machine is one of the best classifiers for the prediction of protein subcellular localization [5, 9, 10, 14, 17, 22], in this study, we focus on obtaining a higher prediction accuracy on the premises of simple feature extraction method and support vector machine to predict the subcellular localization of the apoptosis protein, therefore, finding an efficient approach to optimize the traditional sequence-based feature is the key problem to be solved in this paper.

In the study, we proposed a sparse coding method combined with traditional sequence feature extraction algorithm to extract low-level features of the apoptosis protein sequence, using multilayer pooling based on different sizes of dictionaries to integrate the local and holistic features of the sparse representation. Then the support vector machine was used to complete the final prediction. Given that our adopted benchmark data sets are unbalanced which may influence the classification effects of support vector machine [37], we used an oversampling approach to balance the data sets in the study. Compared with other experimental results with the same support vector machine classifier, the experimental results show that the proposed method can not only simplify the feature extraction process and reduce the time and space complexity of the classifier but also reflect the sequence features more comprehensively and improve the classification performance. The detailed descriptions are shown in the following sections.

## 2. Materials and Methods

### 2.1. Datasets

Two widely used benchmark data sets are adopted in this study: ZD98 and CH317, respectively. The data set ZD98 was constructed by Zhou and Doctor [21]. There are 98 apoptosis protein sequences divided into four kinds of subcellular locations, which are cytoplasmic proteins (Cy), mitochondrial proteins (Mi), membrane proteins (Me), and other proteins (Other). The data set CH317 was constructed by Chen and Li [23] and contains a total of 317 apoptosis protein sequences, in 6 classes of subcellular locations that are secreted proteins (Se), nuclear proteins (Nu), cytoplasmic proteins (Cy), endoplasmic reticulum proteins (En), membrane proteins (Me), and mitochondrial proteins (Mi). Considering that the above data sets are old, we update ZD98 and CH317 data sets with reference to Wang et al. [38] and remove some of the duplicates and error sequences. The specific method is not repeated here. After processing, there were 96 protein sequences remaining in ZD98 data set and 314 protein sequences remaining in CH317 data set. All protein sequences in the above two data sets are from the latest version of the UniProt database (Release 2018_12), and the number of protein sequences in each class of 2 data sets is shown in Table 1.

### 2.2. Feature Extraction

**Figure 1:**
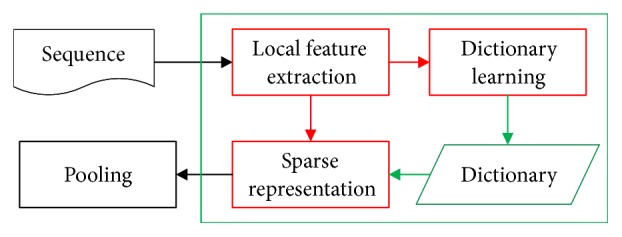
The flow of feature extraction process.

In order to set up a more accurate mapping relationship between each protein sequence and its corresponding feature vector, multilayer sparse coding was introduced in this study to find the most essential feature of original protein sequence based on simple feature extraction method. The algorithm mainly includes the following steps: local feature extraction, sparse coding, and pooling. And the process of sparse coding is divided into 2 sections: dictionary learning and sparse representation. Firstly, the protein sequence is segmented into some fragments, and the traditional protein feature extraction algorithm will be used to extract the features of these fragments, which could be applied for the step of dictionary learning. Then these local features are trained to construct a dictionary and the feature representation of original sequence would be sparsely reconstructed by it. The mean pooling is used to reduce the dimensions of the feature matrix, and finally the pooled vectors based on different dictionary sizes would be integrated as the ultimate features of protein sequences. The flow chart of extraction progress is shown in Figure 1.

#### 2.2.1. Local Feature Extraction

Before the step of sparse coding, it is necessary to extract the local features of protein sequence to constitute a training sample set for dictionary learning. Since every protein sequence has the different length and the critical features may be distributed in different positions of the sequence, in this paper, we adopted sliding window segmentation method inspired by Noor to cut all the protein sequences into pieces [39], generating a number of sequence fragments afterwards. The size of sliding window represents the segment length of each protein sequence, and the reference formula is

(1) Lmin=min⁡L1,L2,…,Lnum,Lmin2≤s≤Lmin,  m∈Z

where *L*_1_, *L*_2_,…, *L*_*num*_ represent the length of each protein sequence in the whole data set, *L*_*min*_ is the shortest sequence of it, and *s* is the size of sliding window, which indicates that the value of segment length is between *L*_*min*_/2 and *L*_*min*_, and the exact value will be selected by the experimental experience.

After the step of segmentation, the existing sequence feature extraction method is used to statistically analyze the component information of sequence fragments and to transform the character sequences into numerical vectors as the local features of the protein. Effective feature extraction method can remarkably increase the final prediction accuracy. Nakashima and Nishikawa [47] firstly associated the amino acid composition (AAC) with the prediction of protein subcellular location in 1994. The AAC coding method was proposed to count the occurrence frequency of each amino acid in the protein sequence, described as follows:

(2) $$\begin{array}{c} P_{AAC}={\left[\;f_{1}f_{2}f_{3}\ldots f_{20}\;\right]}^{T} \end{array}$$

where *f*_1_, *f*_2_, *f*_3_,…, *f*_20_ represent the number of each amino acid in the protein sequence, respectively and the specific explanation is

(3) $$\begin{array}{c} f_{u}=\frac{1}{L}\overset{L}{\underset{i=1}{\sum }}F_{i},\;F_{i}=\left\{\begin{array}{cc} 1, & \text{if }R_{i}=A\left(u\right)\\ 0, & \text{if }R_{i}\neq A\left(u\right) \end{array}\right. \end{array}$$

*L* represents the length of each protein sequence, that is, the total number of all the amino acid residues contained. Firstly, 20 amino acids are numbered from 1 to 20, and *f*_*u*_  (*u* = 1,2, 3,…, 20) describes the frequency of corresponding number appeared in the sequence. *R*_*i*_ represents each amino acid residue in original sequence, and *A*(*u*) represents the amino acid residue which corresponds to the number *u*.

By using AAC to calculate the fragment features of protein sequence* P*, we can obtain a feature matrix for each original protein sequence constituted by all the AAC features of corresponding fragments. The matrix is shown in

(4) $$\begin{array}{c} V=\left[\begin{array}{cccc} v_{11} & v_{1n} & \ldots & v_{1n}\\ v_{21} & \ldots & \ldots & v_{2n}\\ \vdots & \ldots & \ldots & \vdots \\ v_{m1} & v_{m2} & \cdots & v_{mn} \end{array}\right] \end{array}$$

where *m* represents the number of fragments cut by a protein sequence, *n* is the feature dimension processed by AAC algorithm, and *v*_*mn*_ represents the probability of occurrence of different amino acid residues. At this time, *n* is 20. Each row of the matrix represents the feature vectors of different sequence fragments in a protein sequence. Generally we choose some of the fragment features as the local features to construct the dictionary, in this paper; since the number of fragments obtained is not very large, in order to get a better feature representation in spar coding, we chose the local features of all the sequence fragments to form a training sample set *X* = [*x*_1_, *x*_2_, *x*_3_,…, *x*_*N*_] for dictionary learning, where *x*_*i*_ ∈ *R*^*n*^  (*i* = 1,2, 3,…, *N*), *x*_*i*_ represents the feature vector of different protein sequences, that is, the vector in each row in *V*, and *N* is the number of fragments belonged to all of the protein sequences in the data set.

#### 2.2.2. Sparse Coding

Sparse coding is a branch of deep neural networks, and it contains 2 main steps: dictionary learning and sparse representation, respectively [48]. It can extract the detailed features of original data set and decompose the input sample set into a linear combination of multiple primitives. The coefficients of the primitives are the features of input sample. The description can be formulated as

(5) $$\begin{array}{c} X=UD \end{array}$$

where *X* is the matrix of training sample composed by fragment features; *D* = [*d*_1_, *d*_2_, *d*_3_,…, *d*_*K*_] ∈ *R*^*K∗n*^ is the primitive matrix named the dictionary, *d*_*i*_ represents the feature elements of dictionary, *K* is the size of dictionary, *n* is feature dimension* 20* processed by AAC algorithm,; *U* = [*u*_1_, *u*_2_, *u*_3_,…, *u*_*N*_] ∈ *R*^*N∗K*^ is the sparse representation of original sample, and *u*_*i*_ represents the sparse coefficient of the i-th feature block in the sparse feature space, that is, the projection of *x*_*i*_ in sparse feature space. *N* is the number of fragments belonged to all of the protein sequences in the data set. The solution of dictionary *D*  can be expressed as

(6) minD,U ∑i=1Nxi−Dui22s.t ui0≪T0,  i=1,2,3,…,N.

where ‖∙‖_2_ represents *L*_2_ norm of a vector and ‖∙‖_0_ is *L*_0_ norm of a vector. The constraint in formula above means that the number of nonzero elements in *u*_*i*_ needs to be less than or equal to *T*_0_, which is preset and related to the sparse rate. Equation (6) is essentially a nonconvex optimization problem. There are mainly two common solutions for it: the first is to transform it into a convex optimization problem to relax the constraint of equation and then transforms it into the following form:

(7) minD,U ∑i=1Nxi−Dui22+λui1.

where *λ* is the balance factor and ‖∙‖_1_ represents *L*_1_ norm of a vector. Equation (7) can usually be solved by regression algorithm, such as LASSO [49]. The second is to solve it by using the heuristic greedy algorithm [50]. The algorithms for the second solution are MOD and K-SVD [51]. In this study, in view of the efficiency and operability of the algorithm, we choose K-SVD as our solution to learn the dictionary; that is, the second solution.K-SVD is an expansion of K-means algorithm proposed by Aharon and Elad [52]. It adopts the method of iterative alternating learning and uses the singular value decomposition to perform *K* times iterations to optimize the primitives of dictionary, which can better fit the original data. K-SVD is mainly divided into the following steps:

1. Initialize the dictionary *D*, and set the terminal condition of iteration;
2. Fix *D*, solve the sparse representation *U*;
3. Fix *U*, solve the dictionary *D*;
4. Perform steps (2) and (3) alternately until the end of the iteration.

After obtaining the dictionary, the orthogonal matching pursuit (OMP) algorithm is used to complete the sparse representation of the fragment features of the original protein sequence [53]. The basic theory of OMP is to select one of the most matching primitives from the dictionary to perform a sparse approximation with the primitives of original samples and to obtain the residual between them. Then, it continues to select the next proper primitive which is best matched with this signal residual and iterates in this way over and over until the residual and sparse rate meets the fixed terminal conditions. Samples can be approximately presented by a linear combination of these derived primitives. All primitives selected in each process must be orthogonalized first, which would make the convergence speed faster [54]. Constituting the sparse features of all the encoded fragments, we can obtain an *m∗K* sparse matrix *Z* to represent the feature of each protein sequence, where *m* is the number of sequence segments in each sequence and *K* is the size of dictionary, that is, the sparse representation of a protein sequence.

#### 2.2.3. Multilayer Pooling

The dimension of the feature matrix obtained by sparse coding is very high, if we expand it directly, the huge data volume will cause redundant space and time complexities of classification, and it is prone to overfitting. Therefore, it is necessary to reduce the dimensions of the feature matrix. The method of pooling can map the collection of feature vectors into a single vector. There are two different common pooling methods that are the max pooling and mean pooling, respectively. The aggregation statistics of features in different positions can extract the effective information and reduce the calculated amount of numerical matrix [55]. Max pooling takes the maximum value of the feature points in the neighborhood and retains the edge information of the feature matrix more, while mean pooling takes the average value of the feature points in the neighborhood and more to extract the background information [56]. Given that the characters of sequence data are different from images, we chose the mean pooling as the final dimension-reduced method. The formula is shown as follows:

(8) Z=z1,z2,z3,…,zKTzi=meanzi1,zi2,zi3,…zim

where *i* = 1,2, 3,…*K*, *z*_*i*_ being obtained by averaging the *m* elements in the i-th row of the matrix *Z*. After being processed by mean pooling, each protein sequence is represented as a *K* dimensional feature vector, *K* is the size of dictionary.

**Figure 2:**
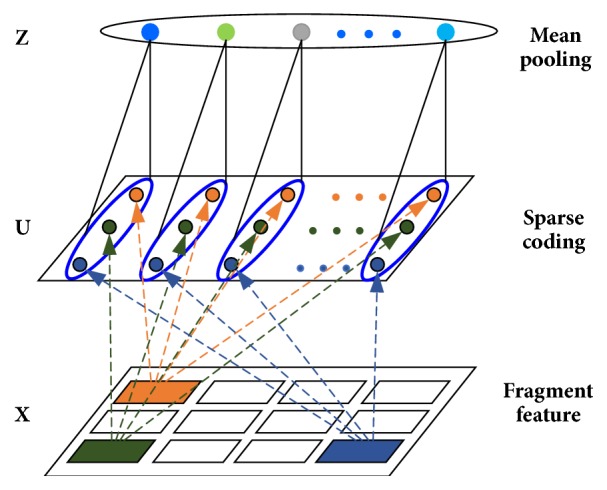
The progress of multilayer pooling.

In order to obtain a more overall feature representation of original protein sequence, multilayer pooling based on different sizes of dictionary is performed, and several pooling results will be integrated to help extract the local and holistic features severally. The specific description is as follows: in the process of sparse coding, the values of dictionary sizes are set to *K*_1_, *K*_2_, and *K*_3_ respectively; thus 3 different levels of dictionary could be obtained by K-SVD algorithm. Then the OMP algorithm is used to complete the sparse representation of fragment features based on different dictionary sizes, and the sparse features are combined to obtain the feature matrix of original sequence. Finally the sparse matrix will be mean pooled to extract different levels of feature vectors. The vectors in each pooled block are concatenated together to obtain a *K*_1_ + *K*_2_ + *K*_3_ dimensional vector as the final feature representation. In this paper, the values of *K* were set to 30, 50, and 70, respectively, generating a 150 dimensional vector to be selected by principal component analysis (PCA) and sent to the classifier for prediction. The general descriptions of spare coding and pooling can be shown in Figure 2.

### 2.3. Oversampling Method

Since the data sets used in this paper are not balanced, which may cause the low accuracy of prediction, we referred to some similar papers used the oversampling to balance the data set [16, 30, 43]. In order to further illustrate the effect of our method, a simple oversampling method called synthetic minority oversampling technique (SMOTE) was applied in the study to decrease the imbalance of our data set. SMOTE is a classical oversampling method proposed by Chawla et al. [57]. It is widely used for its good classification effect and simple operation. The basic principle of SMOTE algorithm is to synthesize new minority samples between a few neighbouring samples and to reduce the imbalance of the data distribution. The details are as follows:

(1) For each sample *X* in the class of smaller number of data set, calculate the Euclidean distance from other samples in the minority class to obtain the *K* nearest neighbor samples.

(2) Assuming that the sampling magnification is *N*, for each of the few classes of samples *X*, *n*(*K* > *n*) samples are randomly selected from their *K* nearest neighbor samples and these *n* samples are recorded as *y*_1_, *y*_2_, *y*_3_, … , *y*_*n*_.

(3) According to the following, combine each sample *X* with *n* samples to perform random interpolation operations to synthesize *n* interpolated samples *Pi*:

(9) $$\begin{array}{c} Pi=X+rand\left(\;0,1\;\right)*\left(\;yi-X\;\right),\;i=1,2,3,\ldots ,n \end{array}$$

where rand(0,1) represents a random number within (0,1) and *yi* represents the i-th nearest neighbor sample of *X*.

(4) Finally, the interpolated sample *Pi* is added to the original sample set to form a new sample set.

The imbalance degree of the data set determines the value of *N*, and the imbalanced level (IL) between majority and minority of the data set is calculated according to

(10) $$\begin{array}{c} N=round\left(\;IL\;\right) \end{array}$$

where *round*(*IL*)  represents the value obtained by rounding up IL. Through the above interpolation operation, the majority and the minority samples can be effectively balanced to improve the accuracy of classification. In this study, the minority classes of 2 data sets are balanced by SMOTE, and the processed results after are as Table 2.

### 2.4. Classifier and Performance Measures

In order to facilitate the comparison with other feature extraction algorithms, we used support vector machine (SVM) as the classification model in this study. After the feature extraction of protein sequences, the universal package of LIBSVM developed by Lin was applied to construct the SVM multiclass classifier [58]. The Jackknife test was also adopted to examine the effectiveness of classifier in our experiment. Jackknife test has the least arbitrary that can always yield a unique result for a given benchmark dataset [59]. Furthermore, in order to have a more comprehensive evaluation, sensitivity (Se), specificity (Sp), Matthew's correlation coefficient (MCC), and the overall accuracy (OA) over the entire data set are applied as the evaluation index [20, 21, 60]. These parameters are detailed in

(11) Se=TPTP+FN

(12) Sp=TNTN+FP

(13) MCC=TP∗TN−FP∗FNTP+FN∗TP+FP∗TN+FN∗TN+FP

(14) OA=∑i=1kTPiN

where* TP*,* TN*,* FP,* and* FN* are the number of true positives, true negatives, false positives, and false negatives, respectively; *N* is the total number of protein sequences and *k* is the class number.

### 2.5. Parameters Selection

**Figure 3:**
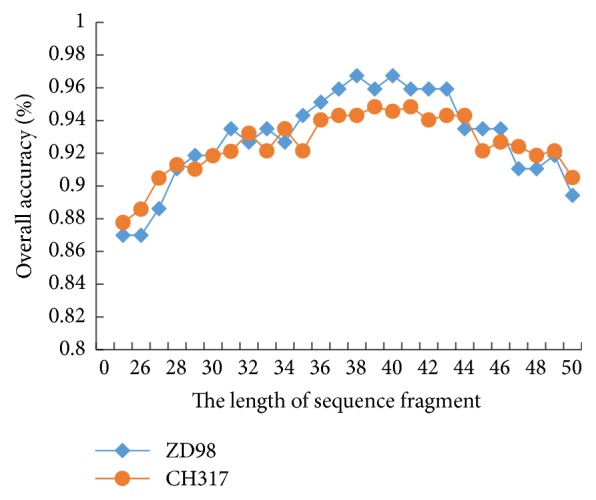
The prediction accuracy based on different lengths of sequence fragments.

There are two key parameters in this study. One is the length of sequence fragment in the local feature extraction. The shortest protein sequence length in the data set is 50, and the fragment length is selected between 25 and 50.  Figure 3 shows the prediction accuracy of the data set ZD98 and CH317, respectively, when taking different slice lengths.

As shown in Figure 3, when the sequence length is between 35 and 40, the prediction accuracies on the data sets ZD98 and CH317 are the highest and tend to be stable, and the current length is the optimal value. The optimal values for the two data sets used in this study are 35 and 40, respectively.

**Figure 4:**
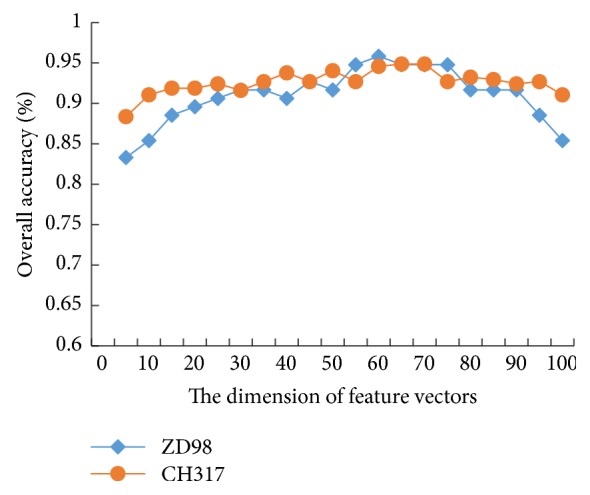
The prediction accuracy based on different dimensions.

When using PCA to select the final feature vectors, the setting of dimension* D* has an effect on the accuracy of the entire algorithm. The more dimensions are selected and the more features are included, but the training time of the classifier may be too long. The smaller the dimension is, the more likely it is to lose some truly meaningful features and affect the classification effect. Therefore, an optimal *D* needs to be sought through experiments.  Figure 4 shows the prediction accuracy corresponding to the different *D* taken by the data sets ZD98 and CH317 during the feature selection of PCA.

As shown in Figure 4, when the dimension of the feature vector is low, the prediction accuracy of two data sets is relatively low. When the dimension is higher than a certain value, the prediction accuracy is also reduced. When the dimension is between 60 and 70, the prediction accuracies on the data sets ZD98 and CH317 are the largest and tend to be steady, and the current *D* is the optimal value. The optimal values for the two data sets used are 60 and 65, respectively.

## 3. Result and Discussion

The prediction results of our experiments by Jackknife on the data sets ZD98 and CH317 are listed in Tables 3, 4, and 5. To further illustrate the effectiveness of our method, the prediction results in each subcellular location of two data sets are also listed in Tables 3–5, which are sensitivity, specificity, correlation coefficient, and overall accuracy, respectively.

It can be seen from Table 3 that the method has obtained good experimental results on both two data sets, and the total accuracies rates are 96.7% and 94.8%, respectively. The experiment proves that the method can effectively increase the accuracy of the prediction of protein subcellular localization. At the same time, in order to facilitate the comparison with other methods, we have listed some experimental results based on some improved algorithms of protein sequence feature extraction in the past several years.

In Tables 4 and 5, DCC_SVM comes from Liang [40], by using detrended cross-correlation coefficient(2016); OF_SVM comes from Zhang [41], by using *λ*-Order Factor and principal component analysis(2017); DE_SVM comes from Liang [42], by fusing two different descriptors based on evolutionary information(2018); BOW_SVM comes from Zhao [24], by using bag of words(2017); GA_SVM comes from Liang [17], by using geary autocorrelation and DCCA coefficient(2017); OA_SVM comes from Zhang [43], by using oversampling and pseudo amino acid composition(2018); IAC_SVM comes from Zhang [44], by using integrating auto-cross correlation and PSSM(2018); EI_SVM comes from Xiang [45], by using evolutionary information(2017); CF_SVM comes from Chen [46], by using a set of discrete sequence correlation factors(2015); all the methods use SVM as the final classifier.

It can be seen from Table 4 that the result on the data set ZD98 has a maximum improvement of the overall prediction accuracy, increasing by about 6 to 8 percentage points compared with traditional protein sequence feature extraction algorithms such as DCC_SVM, OF_SVM, and DE_SVM. In the subcellular class of cytoplasmic proteins, the prediction accuracy rate is 100%, which means that all the sequences in this class are predicted correctly, and the overall prediction accuracy is better than other methods as well. Compared the experimental results with other improved feature extraction algorithms such as BOW_SVM, GA_SVM, and OA_SVM, the accuracy on the same data set is also improved by about 3 to 5 percentage points. Experiments show that the proposed method indeed provides a better source of information for protein sequences and have significant advantages than other similar feature extraction methods. From the comparison in Table 5, we can see that the prediction result on mitochondrial proteins of data set CH317 is up to 96.4%, which is about 4.1% to 14% higher than other algorithms. The accuracy rate in the class of Nuclear has also increased by 14.2% maximally, improving the total prediction accuracy by 3.3 to 4.3 percentage points compared with the improved algorithms such as IAC_SVM, EI_SVM, and CF_SVM, which further demonstrates that the method can optimize the underlying features of the sequence and effectively improve the prediction accuracy of apoptosis protein subcellular localization. Compared with the traditional protein sequence feature extraction and their improved methods, the time complexity of our algorithm is not only low but can also achieve better results based on the simple AAC feature. The background information of the feature representation can also be extracted by mean pooling and comprehensively reflect the distribution of sequence features more, as well as improving the classification accuracy.

## 4. Conclusions

Prediction of apoptosis protein subcellular localization has always been the hotspot of bioinformaticians all over the world. Based on the traditional protein sequence feature extraction algorithm AAC, this paper introduced sparse coding to optimize sequence features and proposed a feature fusion method based on multilevel dictionary. The main contribution includes firstly using sliding window segmentation to extract the sequence fragments of protein sequences, and the traditional feature extraction algorithm was used to encode them. Then the K-SVD algorithm was used to learn the dictionary, and the sequence feature matrix was sparsely represented by the OMP algorithm. The feature representation based on different sizes of dictionaries is mean-pooled to help extract the overall and local feature information. Finally the SVM multiclass classifier is used to predict the subcellular location of the proteins. Experiments show that the proposed method can obtain better results in the prediction success rate of most subcellular classes and have important guiding significance for improving the feature expression of traditional apoptosis protein sequence feature extraction algorithms. Generally speaking, it is a relatively effective method for predicting the subcellular localization of apoptosis proteins.

## Figures and Tables

**Table 1:** Numbers of protein sequences in different class of 2 datasets.

Dataset	Number of sequences in each class						Total
ZD98	Cy	Me	Mi	Other			
	43	30	13	12			98
CH317	Cy	En	Me	Mi	Nu	Se	
	112	47	55	34	52	17	317

**Table 2:** Numbers of protein sequences in different class of 2 datasets.

Dataset	Number of sequences in each class						Total
ZD98	Cy	Me	Mi	Other			
	43	30	26	24			123
CH317	Cy	En	Me	Mi	Nu	Se	
	112	47	55	51	52	51	368

**Table 3:** The experimental results of data sets.

Dataset	Jackknife test(%)				
	Location	Sn(%)	Sp(%)	MMC(%)	OA(%)
ZD98	Cy	100	95.6	95.9	96.7
	Me	96.7	96.7	95.2	
	Mi	92.3	96.0	86.4	
	Other	95.9	95.8	90.5	
CH317	Cy	95.5	93.8	90.9	94.8
	Me	93.6	92.7	91.1	
	Mi	96.4	94.6	96.7	
	Se	94.1	92.3	83.4	
	Nu	94.2	92.5	89.6	
	En	94.1	90.5	91.5	

**Table 4:** Comparison of the accuracy of ZD98 data set.

Methods	Jackknife test(%)				
	Cyto	Memb	Mito	Other	OA(%)
DCC_SVM [40]	93.0	86.7	92.3	75.0	88.9
OF_SVM [41]	97.7	86.3	92.3	66.7	90.8
DE_SVM [42]	95.4	93.3	76.9	83.3	90.8
BOW_SVM [24]	97.7	92.9	76.9	83.3	91.7
GA_SVM [17]	95.4	90.0	92.3	83.3	91.8
OA_SVM [43]	95.3	88.9	97.4	91.7	93.2
Our	100	96.7	92.3	95.9	96.7

**Table 5:** Comparison of the accuracy of CH317 data set.

Methods	Jackknife test(%)						
	Cyto	Memb	Mito	Secr	Nucl	Endo	OA(%)
DCC_SVM [40]	91.1	92.7	82.4	76.5	80.0	93.6	88.3
GA_SVM [17]	92.9	89.1	82.4	76.5	84.6	93.6	89.0
BOW_SVM [24]	94.6	87.3	82.4	82.4	84.3	91.5	89.2
IAC_SVM [44]	96.4	94.5	82.4	76.5	80.8	93.6	90.5
EI_SVM [45]	94.6	95.7	92.7	82.4	90.4	70.6	91.1
CF_SVM [46]	96.4	90.9	92.3	95.7	82.4	64.7	91.5
Our	95.5	93.6	96.4	94.1	94.2	94.1	94.8

## Data Availability

The data used to support the findings of this study is available from the corresponding author upon request, and you can also find it from https://github.com/Multisc/Multi_sc_subloc.
